# Linking Expression of Fructan Active Enzymes, Cell Wall Invertases and Sucrose Transporters with Fructan Profiles in Growing Taproot of Chicory (*Cichorium intybus*): Impact of Hormonal and Environmental Cues

**DOI:** 10.3389/fpls.2016.01806

**Published:** 2016-12-05

**Authors:** Hongbin Wei, Anja Bausewein, Heike Steininger, Tao Su, Hongbo Zhao, Karsten Harms, Steffen Greiner, Thomas Rausch

**Affiliations:** ^1^Plant Molecular Physiology, Centre for Organismal Studies Heidelberg, Heidelberg UniversityHeidelberg, Germany; ^2^Co-Innovation Center for Sustainable Forestry in Southern China, College of Biology and the Environment, Nanjing Forestry UniversityNanjing, China; ^3^College of Horticulture, South China Agricultural UniversityGuangzhou, China; ^4^ZAFES, Südzucker AG Mannheim/OchsenfurtObrigheim, Germany

**Keywords:** *Cichorium intybus*, fructan metabolism, source-sink relationship, phytohormones, environment, leaf blade, petiole, taproot

## Abstract

In chicory taproot, the inulin-type fructans serve as carbohydrate reserve. Inulin metabolism is mediated by fructan active enzymes (FAZYs): sucrose:sucrose 1-fructosyltransferase (1-SST; fructan synthesis), fructan:fructan-1-fructosyltransferase (1-FFT; fructan synthesis and degradation), and fructan 1-exohydrolases (1-FEH1/2a/2b; fructan degradation). In developing taproot, fructan synthesis is affected by source-to-sink sucrose transport and sink unloading. In the present study, expression of FAZYs, sucrose transporter and CWI isoforms, vacuolar invertase and sucrose synthase was determined in leaf blade, petiole and taproot of young chicory plants (taproot diameter: 2 cm) and compared with taproot fructan profiles for the following scenarios: (i) N-starvation, (ii) abscisic acid (ABA) treatment, (iii) ethylene treatment (via 1-aminoyclopropane-1-carboxylic acid [ACC]), and (iv) cold treatment. Both N-starvation and ABA treatment induced an increase in taproot oligofructans. However, while under N-starvation this increase reflected *de novo* synthesis, under ABA treatment gene expression profiles indicated a role for both *de novo* synthesis and degradation of long-chain fructans. Conversely, under ACC and cold treatment oligofructans slightly decreased, correlating with reduced expression of 1-SST and 1-FFT and increased expression of FEHs and VI. Distinct SUT and CWI expression profiles were observed, indicating a functional alignment of SUT and CWI expression with taproot fructan metabolism under different source-sink scenarios.

## Introduction

Chicory (*Cichorium intybus L.*) is a biennial taproot-bearing crop, cultivated for the production of endive and industrial scale extraction of inulin-type fructans. Recently, fructans have gained increasing attention as health-promoting food supplements, e.g., as dietary fiber, low caloric sweeteners or immune-modulators (van Arkel et al., 2013). In chicory, inulin serves as reserve carbohydrate and is accumulated in the taproot during the first year’s growing season. Biosynthesis of inulin, a β-2→1 fructan, is mediated by the enzymes sucrose:sucrose 1-fructosyltransferase (1-SST) and fructan: fructan 1-fructosyltransferase (1-FFT). 1-SST initiates inulin synthesis while 1-FFT serves a dual function, being involved in fructan chain elongation, but also in inulin turnover. Inulin is degraded by fructan 1-exohydrolase, in chicory being encoded by three isoforms, namely 1-FEH1, 1-FEH2a and 1-FEH2b. Noteworthy, short chain oligofructans such as kestose and nystose may also be hydrolyzed by acid invertases (Peukert et al., 2014).

Since sucrose serves as substrate for inulin biosynthesis, its allocation via the phloem from photosynthetically active source leaves to the taproot (heterotrophic sink) during the first year’s growing season is a crucial determinant for fructan accumulation. Among the genes involved in phloem loading in source leaves and phloem unloading in sink organs, sucrose transporters and cell wall invertases (CWI) play pivotal roles in both monocot and dicot plants (Sherson et al., 2003). Thus, during apoplasmic phloem loading, sucrose first exits from mesophyll cells via SWEET proteins, a family of sugar transporters mediating facilitated diffusion into the apoplasmic space (Chen et al., 2010, 2012). The subsequent energy-requiring uphill transport into the sieve element/companion cell (SE/CC) complex requires the action of sucrose/H^+^ symporters and the proton motive force established by an H^+^/ATPase (Williams et al., 2000). Phloem unloading in sink organs may either operate symplasmically via plasmodesmata or apoplasmically. In the latter case, sucrose uptake into sink cells may be mediated again by sucrose/H^+^ symporters and/or, after sucrose hydrolysis via CWI, by hexose/H^+^ cotransporters (Caspari et al., 1994; Williams et al., 2000). In higher plants, sucrose uptake transporters (SUT or sucrose uptake carriers, SUC; the term SUT being used in this paper) are plasma membrane localized sucrose/H^+^ symporters (Ayre, 2011). SUTs are expressed in the phloem along the translocation pathway, serving a function in both phloem loading and unloading (Lemoine, 2000; Kühn et al., 2003; Sauer, 2007).

Upon arrival in sink organs, sucrose may be either irreversibly hydrolyzed into glucose and fructose by invertases or reversibly metabolized to fructose and UDP-glucose by sucrose synthase (Susy) (Ruan et al., 2010; Ruan, 2014), these processes having a profound impact on phloem unloading. Invertases comprise the evolutionarily related vacuolar (VI) and CWI, both existing in several isoforms, and a distinct group of cytosolic invertases (CI) (Sturm and Tang, 1999). High CWI activity creates a sucrose concentration gradient between the SE/CC complex and the surrounding cell wall matrix, thereby promoting phloem unloading and acting as an important regulator for carbon partitioning at whole plant level as demonstrated in mutant (Cheng et al., 1996; Wang et al., 2008) and transgenic studies (Zanor et al., 2009; Ruan et al., 2010).

In chicory, high concentration of sucrose induces expression of fructan synthesis genes while inhibiting the expression of fructan degrading genes (Lothier et al., 2007). Importantly, vacuolar 1-SST may determine sink strength and contribute to yield maintenance upon stress conditions (Van den Ende et al., 1999). Thus, the availability of sucrose in sink organs is one of the decisive factors for fructan synthesis. While CWI activity does not interfere with vacuolar fructan accumulation, VI activity may directly affect fructan synthesis since it competes with 1-SST for substrate, the latter having a much lower affinity for sucrose (Schroeven et al., 2008; Van den Ende et al., 2009). Consequently, during fructan accumulation VI expression is repressed. Source-to-sink transport of sucrose can be affected by many environmental factors, such as water and salt stress, N- and P-deficiency and temperature (Lemoine et al., 2013), these factors also exerting a profound impact on hormonal signaling. For chicory, the expression of FAZY genes in response to several environmental cues including N-deficiency (Kusch et al., 2009) and various abiotic stresses has been the subject of previous studies (De Roover et al., 2000; Michiels et al., 2004; Valluru and Van den Ende, 2008). Conversely, studies linking expression of genes involved in sucrose allocation from source to sink with expression profiles for FAZY genes have not yet been reported.

In a first step to fill this gap, the present study has explored the expression profiles of several SUT and CWI isoforms, as well as VI and Susy, in comparison to FAZY gene expression at whole plant level, i.e., in leaf blades, petioles and taproots of 6-week-old chicory seedlings, exposed to environmental (N-starvation, cold-treatment) of hormonal cues (ABA, ethylene). At this developmental stage, young taproots (diameter ∼2 cm) are in their active growth phase but have already started to accumulate fructans. Therefore, expression profiles of the named genes could be correlated with taproot fructan profiles to monitor whether observed changes of gene expression can be linked to immediate metabolic impact. The results provide a first frame work for future studies on how to modulate the impact of source-to-sink sucrose allocation on fructan accumulation in the developing chicory taproot.

## Materials and Methods

### Plant Cultivation and Treatments

Seedlings of *Cichorium intybus* L. var. Zoom were grown on vermiculite under long day conditions (16 h light/8 h dark) in the greenhouse. For germination, seeds were watered with tap water. Afterward, seedlings were watered every 4 days with nutrient solution (Gamborg B5 medium, including vitamins). Four-week-old seedlings were N-starved for 10 days by omitting the N-source but keeping other macro- and micronutrients identical to Gamborg B5 medium. Phytohormone treatments were performed with 6-week-old seedlings. ABA stock solution was diluted into nutrient solution to a final concentration of 10 μM. For ethylene treatment, seedlings were watered with nutrient solution containing 100 μM 1-aminocyclopropane-1-carboxylic acid (ACC; precursor of ethylene). Control plants were watered with nutrient solution. For cold treatment, seedlings were transferred into the cold room (6°C), and watered with cold water (1°C) to induce immediate stress. Leaf blade, petiole and taproot samples were collected 5, 10, and 24 h after treatment and directly frozen in liquid nitrogen. Organs from four seedlings were pooled for each replicate.

### Bioinformatic Identification of Chicory SUT and CWI Isoforms

To generate a RNAseq database, a mixed RNA sample from chicory hairy roots was sequenced on a HiSeq System (Illumina) by the Deep-Sequencing-Core Facility on Heidelberg Campus ^1^. Subsequent transcriptome assembly was done using SOAPdenovo-Trans^2^. Based on published chicory vacuolar invertase protein sequences (Van den Ende et al., 2002), a tBLASTn search of a chicory RNAseq database identified sequences of three CWI isoforms. A full-length cDNA could be assembled for CiCWI2, while the coding sequences for CiCWI1 and CiCWI3 were incomplete (C-termini missing). Similarly, using the protein sequences of *Arabidopsis* sucrose transporter (AtSUT4, At1g09960) as query, three chicory sucrose transporter sequences were retrieved. Their full-length cDNAs were assembled, and named CiSUT1-3. Protein sequence alignment was generated via Clustal W method in MegAlign (DNASTAR, Inc., Madison, WI, USA). The phylogenetic tree was generated using the Neighbor-Joining method.

### Cloning of Chicory SUT and CWI Isoforms

To confirm the validity of assembled sequences, gene-specific primers were designed to amplify the complete (or partial, in case of CiCWI1 and CiCWI3) open reading frames from cDNA library (see below), prepared from chicory taproot samples. PCR was carried out using 1 μl cDNA (100 ng) as template, 1 μl primers (10 μM), 0.4 μl dNTPs (10 mM), 4 μl 5X buffer, and 0.2 μl Phusion DNA polymerase in 20 μl reaction. PCR conditions were 98°C, 30 s, 1 cycle; 98°C, 15 s; 60°C, 1 min, and 72°C, 1 min, 35 cycles, and 72°C, 5 min, 1 cycle. The PCR products were column-purified and cloned into pJET2.1 vector and further sequenced to confirm accuracy (Eurofins, Germany). Primer pairs for cloning are listed in Supplementary Table S1. Sequences of newly identified CiCWI and CiSUT isoforms were submitted to GenBank. The accession numbers are CiCWI1 (KY029025), CiCWI2 (KY029024), CiCWI3 (KY029026), CiSUT1 (KY029021), CiSUT2 (KY029022), CiSUT3 (KY029023).

### RNA Extraction and cDNA Synthesis

Total RNA was extracted from 80 mg of frozen, homogenized plant organ with the GeneMATRIX Universal RNA Purification Kit (EURX) according to the manufacturer’s instructions. For cDNA synthesis, 1 μg of total RNA was reverse transcribed in 20 μl mixture of oligo(dT) primer, RNase inhibitor, and AMV reverse transcriptase at 42°C for 20 min, followed by 45 min at 50°C (Roboklon).

### Quantitative RT-PCR Expression Analysis

Transcript levels were determined by qPCR with the SYBR Green method on a Rotor-Gene Q (Qiagen). A 15 μl reaction mixture contained the following components: 5 μl cDNA, each 1 μl of each primer (5 μM stock), 1.5 μl buffer, 0.3 μl dNTPs (10 mM each), 5.75 μl water, 0.3 μl JumpStart Taq DNA polymerase and 0.15 μl CYBR Green (1:400 dilution of purchased stock solution of ABsolute^TM^ QPCR SYBR^®^ Green Fluorescein Mix, ABgene). The thermal cycling conditions were 95°C for 6 min, followed by 40 cycles of 95°C for 15 s, 58°C for 30 s, and 72°C for 20 s, followed by a melt cycle with 1°C increment for 5 s each from 56°C to 96°C. The analysis of melting curves, measurement of primer pair efficiencies, and determination of cycle threshold values, including the calculation of the mean normalized expression of the genes, were conducted using the Rotor-Gene Q Series Software Q 2.0.2 (Qiagen) and the Q-Gene software. Gene expression was calculated relative to transcript profiles of two reference genes Actin and RPL19 (Maroufi et al., 2010; van Arkel et al., 2012). Primer efficiency was considered valid when calculated efficiency was between 90 and 110% with 100% as an optimum. Primer pairs for qRT-PCR analysis are listed in Supplementary Table S2. For further validation of reference genes, it was experimentally confirmed that both reference genes were expressed at comparable ratios in different plant organs and not affected by the different treatments (**Supplementary Figure S1**).

### Metabolite Analysis via HPAEC-PAD

Carbohydrate analysis via high-performance anion exchange chromatography (HPAEC) was performed to determine inulin profiles, and to quantify glucose, fructose, sucrose, 1-kestotriose, 1,1-kestotetraose and 1,1,1-kestopentaose. Measurements were operated on a Dionex ICS-3000 system with the Chromeleon 6.60 software (all components from Dionex). The extraction of total soluble carbohydrates was described in detail by Kusch et al. (2009). In brief, aliquots of the final supernatant were dried in a speedvac concentrator (Bachofer, Reutlingen, Germany). Subsequently, sugar pellets were dissolved in HPLC water (VWR Prolabo) to 10 mg/ml. Final sugar concentration of 0.2 mg/ml and 0.5 mg/ml for taproot and shoot organs respectively were subjected to carbohydrate analysis via HPAEC-PAD. For peak identification, glucose (Merck, Darmstadt, Germany), fructose (Applichem), sucrose (Applichem), 1-kestotriose, 1,1-kestotetraose, 1,1,1-kestopentaose (all Wako Chemicals) and RaftilineST (Orafti, Tienen, Belgium) were used as external standards.

### Statistical Analysis of Data

Gene expression profiles via qPCR and oligofructan contents were determined from three independent experiments. Results are presented as means ± SD. Student’s *t*-test was performed to verify the statistical significance between control and different treatments at the different time points. Asterisks represent significant differences (^∗^*P* < 0.05; ^∗∗^*P* < 0.001).

## Results

### Chicory Cell Wall Invertase (CiCWI1-3) and Sucrose Transporter (CiSUT1-3) Isoforms Are Differentially Regulated during Plant Development

To address the developmental expression profile of CWI and SUT isoforms during plant development, a RNAseq database was generated and searched for isoforms not yet available in the public domain. Three chicory CWI isoforms (CiCWI1-3; including two incomplete cDNA sequences) and three SUT isoforms (CiSUT1-3) could be identified (**Supplementary Figures S1** and **S2**). In comparison to VI, all three CWI isoforms contained a proline residue in their cysteine-containing active site, which differentiates CWIs from VIs (Goetz and Roitsch, 1999), whereas CWIs and VIs share the common β-fructosidase motif (NDPD/NG) (**Supplementary Figure S2**). While the sequence of CiCWI2 (accession number: KY029024) encodes a full length protein (566 amino acids), CiCWI1 (accession number: KY029025) and CiCWI3 (accession number: KY029026) present incomplete protein sequences, lacking part of their C-terminal ends. Searching the same chicory RNAseq database (see above), we have identified three full-length SUT isoforms (**Supplementary Figure S3A**): CiSUT1 (accession number: KY029021; 514 amino acids), CiSUT2 (accession number: KY029022; 493 amino acids) and CiSUT3 (accession number: KY029023; 566 amino acids). According to phylogenetic analysis (**Supplementary Figure S3B**), CiSUT1 is related to AtSUC2, both falling into the SUT1 subfamily. CiSUT2 groups with AtSUT4 into the SUT4 subfamily, whereas CiSUT3 exhibits a high sequence similarity to AtSUC3, grouping into the SUT2 subfamily (Kühn, 2003).

**FIGURE 1:**
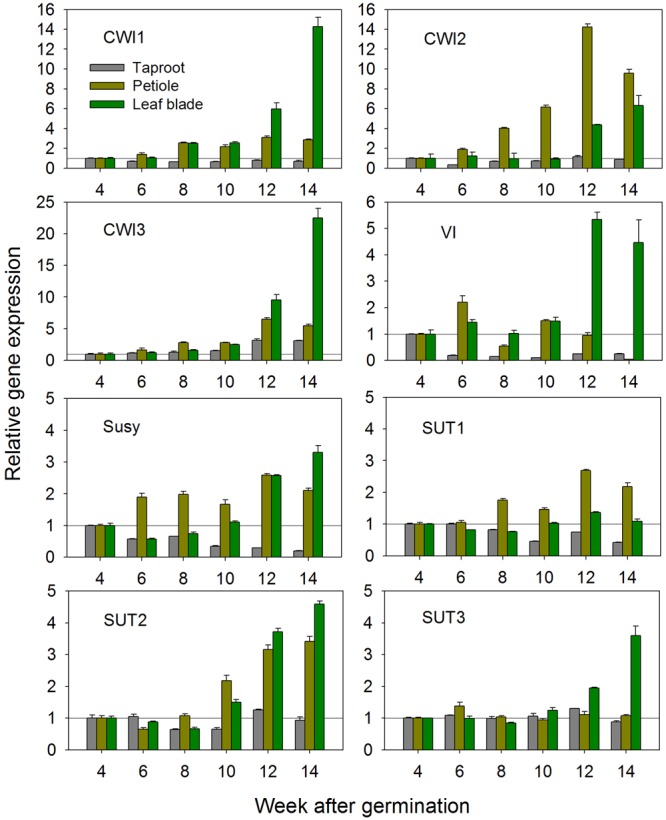
**Transcript levels of cell wall invertase isoforms (CWI1-3), vacuolar invertase (VI), sucrose synthase (Susy) and sucrose transporter isoforms (SUT1-3) in leaf blades, petioles and taproots of chicory seedlings during a 14-week growth period.** Transcript levels were determined by qPCR and normalized against the expression of two reference genes (Actin and RPL19). Transcript levels in different plant organs were expressed relative to the levels of 4-week-old seedlings, which were arbitrarily set to 1 as indicated with a horizontal line.

Based on the newly acquired sequence information, expression of all CiCWI and CiSUT isoforms was determined at transcript level during plant development at 2-week intervals, starting 4 weeks after germination, i.e., the time point when radial root growth initiated taproot development and inulin accumulation. **Figure 1** depicts the developmental changes of gene expression in leaf blades, petioles and taproots. Comparison of CWI expression profiles reveals that CiCWI2 transcript levels are particularly high in petioles throughout plant development, pointing to a specific role in this plant organ. Late during plant development at early leaf senescence, expression of all three CiCWI isoforms is strongly induced in leaf blades, accompanied by induction of VI. Conversely, in taproot the expression of CiCWI isoforms 1 and 2 remains rather stable, with a moderate increase being observed only for CiCWI3. As expected, VI expression in taproot strongly declines after 4 weeks of culture, in agreement with the onset of fructan accumulation, this decline being accompanied by reduced expression of Susy. Expression of CiSUT1-3 remains fairly stable in all organs till about 10 weeks after germination, but at the end of the growing period, CiSUT2 expression is moderately induced in leaf blades and petiole.

Based on these results, 6-week-old seedlings with taproot diameter of approximately 2 cm were chosen for stress and hormonal treatments. These seedlings are in the early exponential growth phase, a stage where VI expression is already strongly reduced reflecting early fructan accumulation, whereas expression profiles for CiSUT and CiCWI isoforms are rather stable, thus minimizing possible interference of stress-induced expression changes with developmentally induced switches.

### Nitrogen Starvation-Induced Expression of 1-SST and 1-FFT in Leaf Blade, Petiole and Taproot Correlates With Fructan Accumulation in Taproot and Petiole-Specific Up-Regulation of CiCWI and CiSUT Isoforms

**FIGURE 2:**
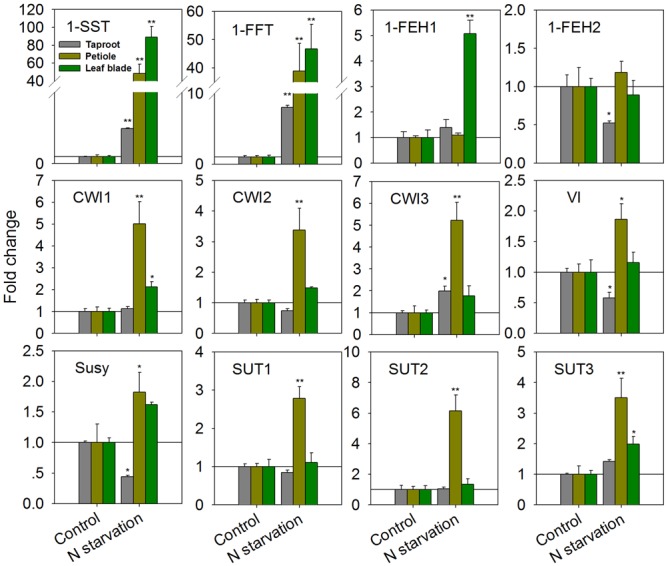
**Impact of nitrogen starvation (10 days) on transcript levels of FAZYs (1-SST, 1-FFT, 1-FEH1 and 1-FEH2), sucrose-cleaving enzymes (CWI1-3, VI, Susy) and sucrose transporter isoforms (SUT1-3) in leaf blades, petioles and taproots of 38-day-old chicory seedlings.** Transcript levels were determined by qPCR, normalized against the expression of two reference genes (Actin and RPL19), and plotted as fold change relative to the control which was set to 1 as indicated with as horizontal line. Bars indicate means ± SD of three independent experiments. Asterisks represent significant differences as determined by Student’s *t*-test (^∗^*P* < 0.05; ^∗∗^*P* < 0.001).

As expected, N-starvation of chicory seedlings for 10 days (starting 4 weeks after germination) resulted in strongly increased transcript levels for 1-SST and 1-FFT in all three organs (**Figure 2**). In the taproot, expression of 1-SST and 1-FFT was induced by 5- and 8-fold, respectively, while induction of both genes was more than 40-fold in the leaf blade and petiole. However, this difference reflects the constitutively lower expression in the latter plant organs (1-SST expression ratio of leaf blade/petiole/taproot controls: 1/2.3/12.4; 1-FFT expression ratio of leaf blade/petiole/taproot controls: 1/2.0/10.9). In the taproot and petiole, transcript levels of 1-FEH1 were not affected, but its expression was significantly up-regulated fivefold in leaf blades (**Figure 2**). Expression of the second fructan 1-exohydrolase isoform 1-FEH2 was not affected in leaf blade and petiole, but was suppressed in taproot. In petiole, N-starvation caused significant up-regulation of all CiCWI isoforms, whereas this effect was only moderate in leaf blades; similarly, expression of all CiSUT isoforms was induced in petioles but not in leaf blades. In taproots, VI and Susy expression were both reduced, in agreement with repressed 1-FEH2 expression and N-starvation induced fructan accumulation (see below).

**FIGURE 3:**
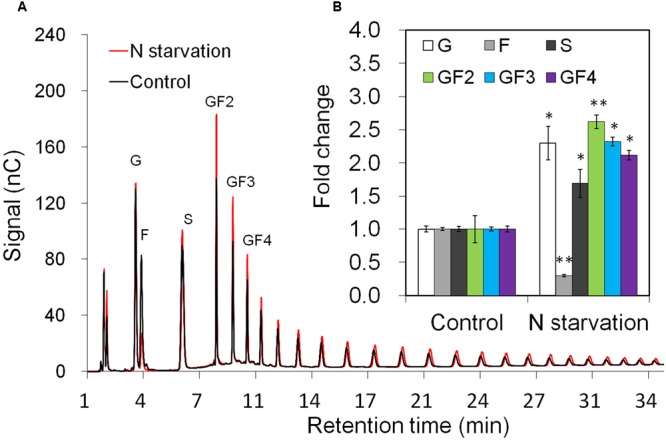
**Impact of nitrogen starvation on fructan composition in taproots of 38-day-old chicory plants: (A)** representative fructan profiles of control and 10 days’ N-starved seedlings; **(B)** quantitative analysis of short chain fructans from control and N-starved seedlings. glucose (G), fructose (F), sucrose (S), 1-kestotriose (GF2), 1,1-kestotetraose (GF3), 1,1,1-kestopentaose (GF4). Each sugar component was plotted as fold change relative to the control. Values are means ± SD of three independent experiments. ^∗^*P* < 0.05; ^∗∗^*P* < 0.001. Carbohydrate measurements via HPAEC-PAD were carried out with a ICS-3000 system and Carbpac PA1 column (Dionex).

Taproot fructan profiles indicated a slight increase after 10 days of N-starvation (**Figure 3A**). For exact quantification, absolute amounts of sucrose, glucose, and fructose as well as oligofructans (GF2 – GF4) were determined (**Figure 3B**). While glucose and sucrose contents were increased, together with up to 2.5-fold increased oligofructans, fructose content was strongly decreased, in agreement with induced inulin synthesis and reduced expression of VI and 1-FEH2.

### Abscisic Acid Induces Expression of 1-FEH1 and 1-FEH2 Throughout the Plant and Up-Regulation of 1-SST and 1-FFT in Leaf Blade and Petiole, Accompanied by Organ-Specific Effects on CiSUT and CiCWI Expression

**FIGURE 4:**
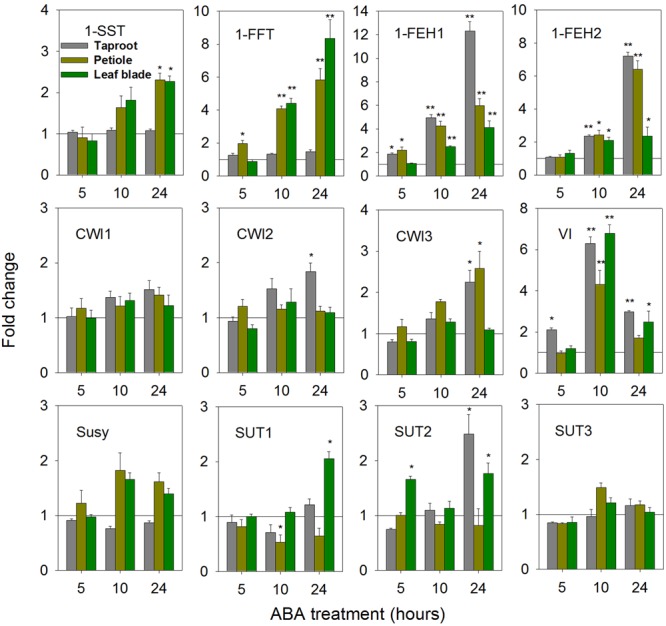
**Impact of abscisic acid (ABA) treatment on transcript levels of FAZYs (1-SST, 1-FFT, 1-FEH1 and 1-FEH2), sucrose-cleaving enzymes (CWI1-3, VI, Susy) and sucrose transporter isoforms (SUT1-3) in leaf blades, petioles and taproots of 6-week old chicory seedlings.** Seedlings were watered with nutrient solution containing 10 μM ABA, while mock plants were watered with nutrient solution only. Leaf blades, petioles and taproots (diameter 2.0 ± 0.3 cm) were harvested at 5, 10, and 24 h following treatment. Transcript levels were determined by qPCR and normalized against the expression of RPL19. Displayed values are means ± SD of three independent experiments. Fold change for ABA treatment was calculated relative to mock samples that were set to 1 as indicated with a horizontal line (fold > 1.0, expression induced; fold < 1.0, expression suppressed). Asterisks represent significant differences as determined by Student’s *t*-test (^∗^*P* < 0.05; ^∗∗^*P* < 0.001).

Abscisic acid is an important stress hormone, being involved in stress-signaling during drought, cold and high salt stress (Raghavendra et al., 2010; Lee and Luan, 2012; Yoshida et al., 2014). ABA-treatment affected FAZY expression in a highly organ-specific manner (**Figure 4**). While in leaf blade and petiole, strong up-regulation of 1-FFT expression correlated with a moderate induction of 1-SST, indicative of fructan biosynthesis, expression of both genes remained unaffected in taproot. Expression of 1-FEH1 and 1-FEH2 showed consistent responses, being up-regulated at whole plant level. Among CWI isoforms, only CiCWI3 expression was significantly induced in petiole and taproot, whereas VI transcripts showed a strong but transient induction upon ABA-treatment. Expression of SUT isoforms CiSUT1 and CiSUT2 revealed differential responses in taproot and leaf blade, respectively, whereas expression of CiSUT3 was not affected by ABA-treatment.

**FIGURE 5:**
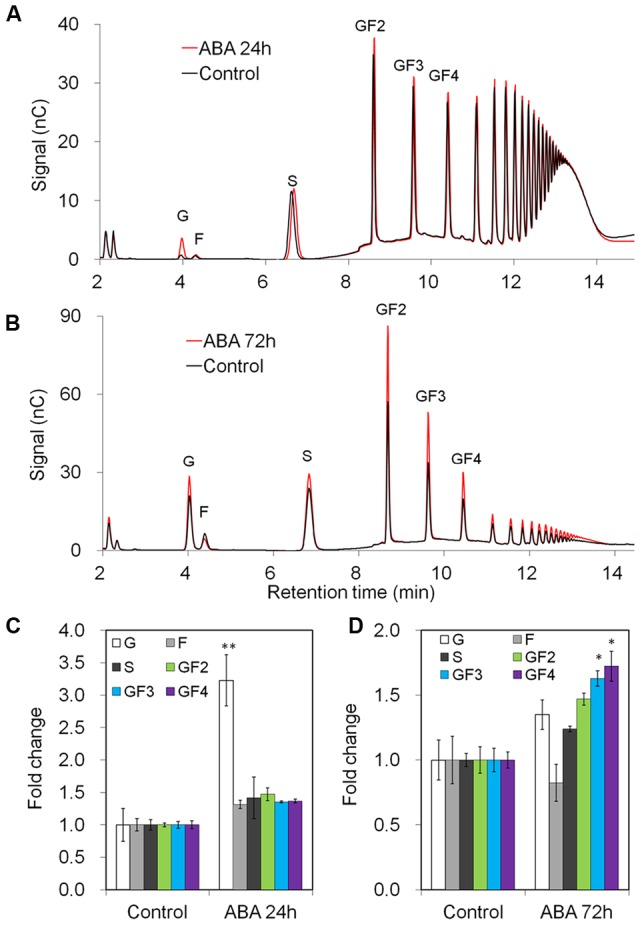
**Impact of ABA treatment on fructan composition in taproots of 6-week-old chicory plants: (A,B)** representative fructan profiles of control and ABA-treated seedlings for 24 and 72 h, respectively; **(C,D)** quantitative analysis of short chain fructans from control and ABA-treated seedlings for 24 and 72 h, respectively. glucose (G), fructose (F), sucrose (S), 1-kestotriose (GF2), 1,1-kestotetraose (GF3), 1,1,1-kestopentaose (GF4). Each sugar component was plotted as fold change relative to the control. Values are means ± SD of three independent experiments. ^∗^*P* < 0.05; ^∗∗^*P* < 0.001. Carbohydrate measurements via HPAEC-PAD were carried out with a ICS-3000 system and Carbpac PA1 column (Dionex).

After 24 h of ABA-treatment, taproot fructan profile was barely affected, as shown by quantitative determination of oligofructans (**Figures 5A,C**). Interestingly, glucose content was significantly increased, likely resulting from the strong induction of VI expression (**Figure 4**). Noteworthy, prolonged ABA-treatment to 72 h caused an induction of 1-SST and 1-FFT expression also in taproots, correlating with an increased content of oligofructans (**Supplementary Figure S4, Figures 5B,D**).

### In Taproot, ACC-Mediated Ethylene Formation Represses 1-SST and 1-FFT, While Transiently Inducing 1-FEH1 and VI Expression

**FIGURE 6:**
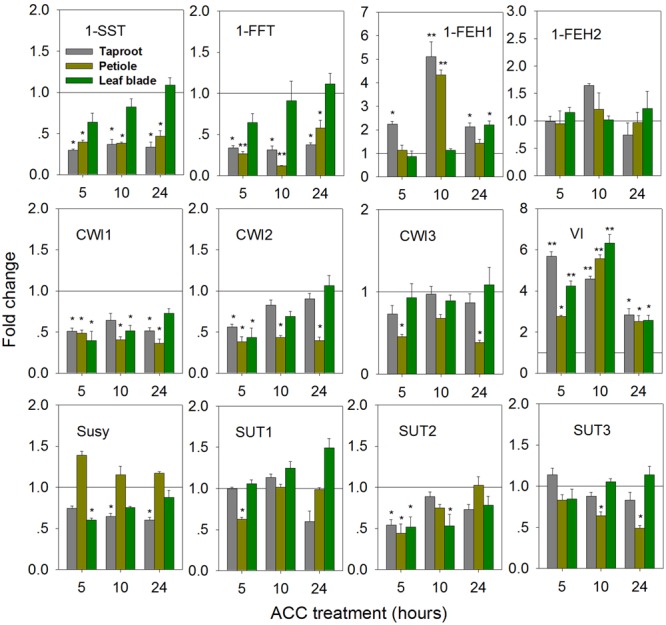
**Impact of 1-aminocyclopropane-1-carboxylic acid (ACC, ethylene precursor) treatment on transcript levels of FAZYs (1-SST, 1-FFT, 1-FEH1 and 1-FEH2), sucrose-cleaving enzymes (CWI1-3, VI, Susy) and sucrose transporter isoforms (SUT1-3) in leaf blades, petioles and taproots of 6-week old chicory seedlings.** Seedlings were watered with nutrient solution containing 100 μM ACC, while mock plants were watered with nutrient solution only. Transcript levels were determined by qPCR and normalized against the expression of RPL19. Displayed values are means ± SD of three independent experiments. Fold change for ACC treatment was calculated relative to mock samples that were set to 1 as indicated with a horizontal line (fold > 1.0, expression induced; fold < 1.0, expression suppressed). Asterisks represent significant differences as determined by Student’s *t*-test (^∗^*P* < 0.05; ^∗∗^*P* < 0.001).

Treatment of seedlings with 100 μM 1-aminocyclopropane-1-acrboxylic acid (ACC), a precursor of ethylene, induced gene expression changes indicative of fructan degradation in the taproot (**Figure 6**). Thus, transcript levels of 1-SST and 1-FFT were reduced in taproot and petiole, accompanied by significantly increased expression of 1-FEH1 and VI, the latter responses being strongest at 10 h of ACC treatment. Noteworthy, 1-FEH2 expression was barely responsive to ACC treatment. Conversely, expression of CiCWI1 and CiCWI2 was reduced throughout the plant, while CiCWI3 expression was only repressed in the petiole. CiSUT2 expression was transiently downregulated at whole plant level, whereas CiSUT1 and CiSUT3 expression was only repressed in the petiole, albeit at different treatment time.

**FIGURE 7:**
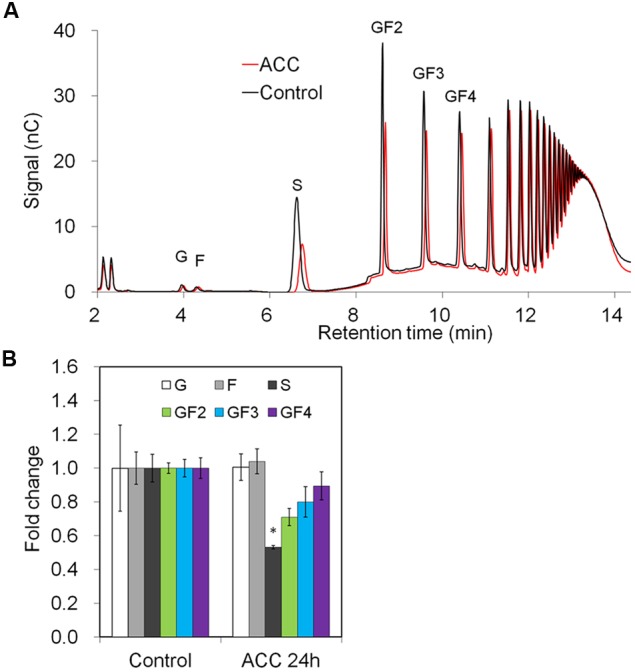
**Impact of ACC treatment on fructan composition in taproots of 6-week-old chicory plants: (A)** representative fructan profiles of control and 24-h-ACC-treated seedlings; **(B)** quantitative analysis of short chain fructans from control and ACC-treated seedlings. Glucose (G), fructose (F), sucrose (S), 1-kestotriose (GF2), 1,1-kestotetraose (GF3), 1,1,1-kestopentaose (GF4). Each sugar component was plotted as fold change relative to the control. Values are means ± SD of three independent experiments. ^∗^*P* < 0.05. Carbohydrate measurements via HPAEC-PAD were carried out with a ICS-3000 system and Carbpac PA1 column (Dionex).

Downregulation of genes involved in phloem sugar transport was mirrored by the significantly decreased sucrose content in the taproots. There was also a trend toward decreased oligofructan levels (GF2-4), but this was not statistically significant probably due to short treatment time (24 h) (**Figures 7A,B**).

### Cold-Induced Fructan Degradation in Taproot: Low Temperature Represses 1-SST and 1-FFT Expression While Strongly Inducing 1-FEH1, 1-FEH2 and VI

**FIGURE 8:**
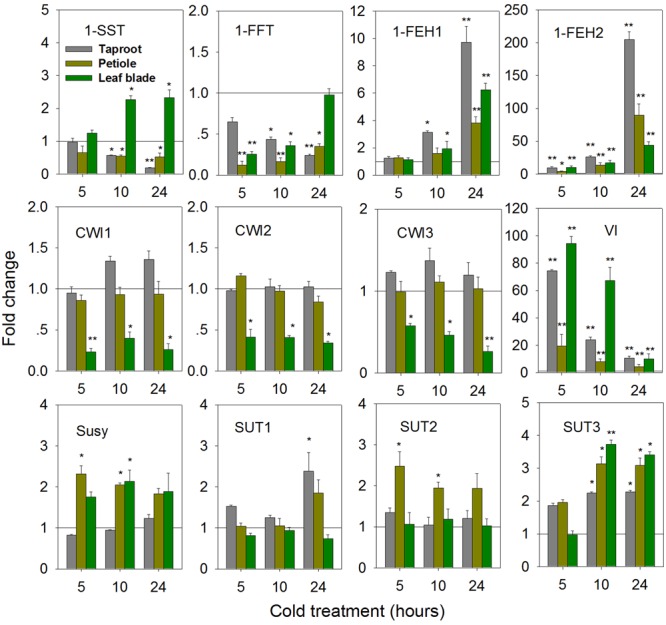
**Impact of cold treatment (6°C) on transcript levels of FAZYs (1-SST, 1-FFT, 1-FEH1 and 1-FEH2), sucrose-cleaving enzymes (CWI1-3, VI, Susy) and sucrose transporter isoforms (SUT1-3) in leaf blades, petioles and taproots of 6-week old chicory seedlings.** Seedlings were transferred to cold room (6°C), while control plants were kept at 25°C. Transcript levels were determined by qPCR and normalized against the expression of RPL19. Displayed values are means ± SD of three independent experiments. Fold change for cold treatment was calculated relative to mock samples that were set to 1 as indicated with a horizontal line (fold > 1.0, expression induced; fold < 1.0, expression suppressed). Asterisks represent significant differences as determined by Student’s *t*-test (^∗^*P* < 0.05; ^∗∗^*P* < 0.001).

While cold-induced fructan degradation in mature taproots is well documented (Van den Ende and Van Laere, 2002; Michiels et al., 2004), the results presented here add the perspective of whole plant level at the state of early taproot development. Compared to control seedlings, expression of 1-SST and 1-FFT was significantly decreased in the taproot and petiole upon cold-treatment (**Figure 8**). This decrease contrasted with strong induction of 1-FEH1, 1-FEH2, and VI at whole plant level. In contrast to the response observed after ABA-treatment, 1-FEH2 was much more responsive to cold than 1-FEH1, showing an up to 200-fold increase of transcript level.

Expression of CiCWI isoforms revealed organ-specific responses to cold-treatment. While in leaf blade transcript levels of CiCWI1-3 were significantly decreased, their expression in petiole and in taproot was barely affected. Likewise, the response of CiSUT expression to cold-treatment indicated pronounced organ specificity. While CiSUT1 was selectively induced in taproot, CiSUT2 was only induced in petiole, whereas CiSUT3 showed increased transcript levels in all three organs.

**FIGURE 9:**
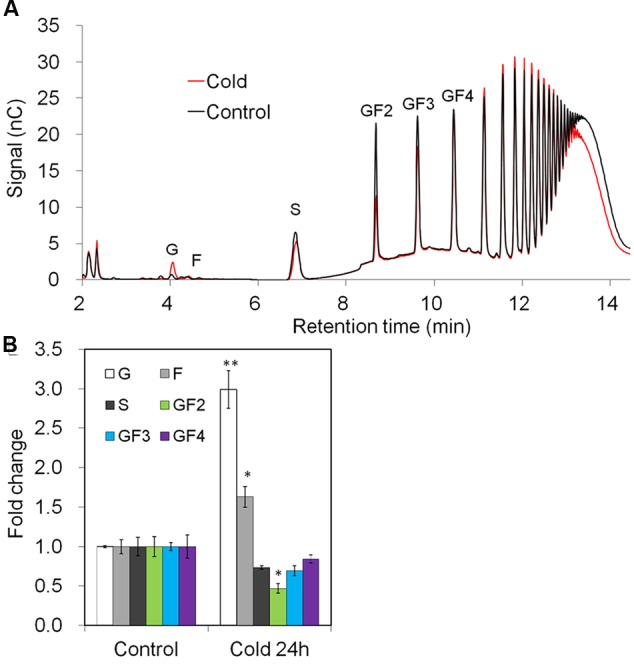
**Impact of cold treatment on fructan composition in taproots of 6-week-old chicory plants: (A)** representative fructan profiles of control and 24- h-cold-treated seedlings; **(B)** quantitative analysis of short chain fructans from control and cold-treated seedlings. Glucose (G), fructose (F), sucrose (S), 1-kestotriose (GF2), 1,1-kestotetraose (GF3), 1,1,1-kestopentaose (GF4). Each sugar component was plotted as fold change relative to the control. Values are means ± SD of three independent experiments. ^∗^*P* < 0.05; ^∗∗^*P* < 0.001. Carbohydrate measurements via HPAEC-PAD were carried out with a ICS-3000 system and Carbpac PA1 column (Dionex).

The observed changes in gene expression upon cold-treatment correlated with changes in taproot oligofructans (**Figures 9A,B**). While fructose and glucose content were prominently increased, in agreement with the strong induction of VI (**Figure 8**), sucrose and oligofructan contents were lowered, however, only 1-kestose content (GF2) was significantly reduced (**Figure 9B**).

## Discussion

This study correlates the expression of fructan active enzymes (FAZYs) with the expression of SUT-type sucrose transporter and CWI isoforms, and links these expression profiles to oligofructan content in early developing taproots of young chicory plants. Combining expression responses under two important environmental cues, i.e., N-starvation and cold-treatment, and two hormonal cues (ABA, ethylene precursor ACC) signaling drought (ABA) and water logging (ACC), respectively, this study provides insight into the dynamic regulation of source-to-sink sugar transport and fructan metabolism, and indicates that at whole plant level petioles display gene expression profiles distinct from leaf blade and taproot, respectively. Although enzyme activities were not determined, previous studies on chicory (Van Laere and Van den Ende, 2002) and on durum wheat (Cimini et al., 2015) have shown that FAZY expression at transcript level largely correlates with corresponding enzyme activities, suggesting that fructan enzymes are mainly controlled at the transcriptional level.

Delivery of sucrose from the leaf blade via the phloem is important for the growth and development of sink organs, like petioles and taproot. In chicory seedlings, approximately 3 weeks after sowing, fructans start to accumulate in the developing taproot (Druart et al., 2001). With the taproot acting as major sink during the first year’s growing season, allocation of photoassimilates to the taproot is a key factor for inulin production. For the present study, the choice of 6-week-old seedlings was based on the results of CiSUT and CiCWI isoform expression profiles (**Figure 1**). This stage represents the early rapid growth phase of massive assimilate allocation from shoot to taproot before the onset of early leaf senescence in mature leaves, which is accompanied by induction of CWI and VI in the leaf blade.

### N-Starvation Imposes Adaptations of Source-Sink Relationship and Fructan Metabolism at Whole Plant Level

In higher plants, N-starvation generally causes accumulation of sugars in leaves, which exert feedback inhibition on photosynthesis (Martin et al., 2002). Simultaneously, allocation of photoassimilates to the root is increased, resulting in a higher root/shoot biomass ratio and alteration of root morphology (Hermans et al., 2006). For non-fructan species, microarray analysis of shoots of N-deficient plants has revealed induction of genes involved in primary metabolism, including starch metabolism, glycolysis and disaccharide metabolism, respectively (Scheible et al., 2004). For chicory, low N-supply was reported to induce 1-SST activity and fructan accumulation (Van den Ende et al., 1999). The increased sucrose content in taproots of N-starved chicory seedlings (**Figure 3B**) might account for the induced expression of fructosyltransferases. A similar effect of sucrose on fructan synthesis has been shown in other fructan-accumulating plant species (Maleux and Van den Ende, 2007; Xue et al., 2011). However, a higher C/N-ratio, rather than high sucrose alone, was shown to be the major determinant for 1-SST and 1-FFT induction in chicory hairy root cultures (Kusch et al., 2009).

By choosing chicory seedlings during early taproot development, the present study provides additional insight into the adaptation to N-starvation at whole plant level. First, 1-SST and 1-FFT are induced throughout the plant (leaf blade, petiole and taproot; **Figure 2**), suggesting that increased fructan synthesis is not restricted to the taproot (**Figure 3**). Second, petioles show a unique reaction pattern in that all CiSUT and CiCWI isoforms are coordinately up-regulated upon N-starvation (**Figure 2**). However, although petiole and leaf blade show a strong induction of 1-SST and 1-FFT expression, this did not result in massive fructan accumulation; in fact, only minor amounts of short chain oligofructans were formed (**Supplementary Figure S5**). Regarding the strong induction of CiSUT and CiCWI isoforms in petioles it may be speculated that local phloem unloading is upgraded. Interestingly, glucose content displays a particularly high level in petioles of control and N-starved seedlings (**Supplementary Figure S5B**). Up-regulation of CiSUT3 in the leaf blade may be required for increased phloem loading. Interestingly, expression of CiCWI isoforms is not only induced in the taproot (CiCWI3), where its expression may support phloem unloading, but also in the leaf blade (CiCWI1), indicative of some futile cycling in the leaf apoplasmic space (i.e., sucrose hydrolysis followed by re-uptake into mesophyll cells).

### In Response to ABA Treatment, Expression of Fructan Exohydrolases in Taproot Correlates With Accumulation of Shorter Chain Oligofructans

ABA signaling plays a vital role in inducing abiotic stress tolerance during water deficit and low temperature (Shinozaki et al., 2003; Zhang et al., 2006; Yoshida et al., 2014). The results reported here indicate that within the first 24 h of ABA-treatment induction of sugar transporters in the leaf blade (CiSUT1&2) and taproot (CiSUT2), concomitant with induction of CiCWI2&3 in the taproot, promote photoassimilate translocation to the taproot (**Figure 4**). Similar results were recently reported for *Arabidopsis* under water deficit, the enhanced assimilate translocation from source to sink being linked with higher expression of sucrose transporter genes in both leaves and roots (Durand et al., 2016).

The observation of simultaneous induction of fructan synthesizing genes within 24 h (**Figure 4**; however, in taproot only after 72 h: **Supplementary Figure S4**) and fructan-degrading FEH genes, together with the increase of oligofructans in the taproot (**Figure 5D**) reflects an ABA-induced rebalancing of fructan pools toward oligofructans. The observed increase in 1-SST and 1-FFT transcripts after ABA treatment of chicory seedlings corroborates the previously reported induction of fructan synthesis via up-regulation of 1-SST expression and enzyme activity (De Roover et al., 2000; see also **Supplementary Figure S6**, confirming induction of fructan synthesis in petioles).

In *Viguiera discolor*, the production of osmoprotective low-DP oligofructans also correlates with concomitant up-regulation of 1-SST and 1-FEHs, reflecting the strategy of oligofructan production via both *de novo* synthesis and inulin degradation (Oliveira et al., 2013). Fructan metabolism has been linked to plant adaption to abiotic stresses (Livingston et al., 2009). In particular, small oligofructans may serve as osmolytes in maintaining osmotic homeostasis, provide direct protection of phospholipid membranes, and act as scavengers of reactive oxygen species (Van den Ende et al., 1998; Valluru and Van den Ende, 2008; Peukert et al., 2014). The strong but transient increase of VI transcript abundance (**Figure 4**) in leaf blade and taproot may affect vacuolar sucrose/hexose ratio, thereby contributing to osmotic adjustment as part of an ABA-induced stress response.

Noteworthy, while the results reported here refer to short and transient changes in ABA signaling, long-term dehydration in chicory may inhibit the activity of fructan synthetic enzyme (1-SST and 1-FFT) and increase 1-FEH activity, resulting in lower inulin production (Vandoorne et al., 2012).

### ACC-Mediated Ethylene Release Induces Gene Expression Change at Whole Plant Level Indicative of Fructan Degradation and Reduced Source-to-Sink Allocation

Ethylene acts as an important modulator of plant growth and development (Wang et al., 2002; Achard et al., 2003). Particular reasons for including the ethylene precursor ACC in the present study were: (i) ethylene is an important stress hormone during water logging (Van Der Straeten et al., 2001; Sairam et al., 2008), (ii) ethylene is known to affect gene expression via ribosome-recruitment under low oxygen pressure as anticipated for the interior of taproot (Juntawong et al., 2014), and (iii) under stress exposure ethylene shows intensive cross talk with ABA signaling (Gazzarrini and Mccourt, 2003; Arc et al., 2013).

ACC treatment resulted in a strong overall repression of fructan biosynthesis genes in petiole and taproot, accompanied by transiently increased expression of 1-FEH1 in these plant organs. Our results are in agreement with responses of fructan associated genes in ryegrass (*Lolium perenne* L.) upon ethylene treatment (Gasperl et al., 2015). During the time course of ACC treatment, up-regulation of 1-FEH1 expression started in the taproot, then extending to petiole and leaf blade; indicating a sequential response due to ACC delivery via the root system. The simultaneous decline of CiSUT and CiCWI expression throughout the plant suggests that assimilate translocation is rather restricted. Interestingly, the strong but transient expression of 1-FEH1 (**Figure 6**) caused only a significant reduction of oligofructans (**Figure 7**). As under biotic and abiotic stress, ABA and ethylene signaling are known to interact (Manavella et al., 2006), it is expected that under field conditions the individual effects observed in the present study are modulated in a complex manner.

### Cold Exposure Induces Fructan Degradation But Stimulates the Expression of SUT-Type Sucrose Transporters Throughout the Plant

For chicory field cultivation, low temperature at the end of the growing season is one of the most important environmental factors negatively affecting inulin yield and quality. However, while a rapid drop in temperature inhibits photosynthesis and results in reduced assimilate allocation to developing sink organs (Ensminger et al., 2006), the reduced inulin content of mature chicory taproots is known to be the result of increased FEH activities induced by cold temperature at the end of the growing season (Van den Ende and Van Laere, 1996; Michiels et al., 2004; van Arkel et al., 2012). In the cold-treated chicory seedlings sucrose transporters showed increased expression in leaf blade, petiole and taproot, indicating that upon short periods of cold-treatment capacity for phloem loading may not be affected (**Figure 8**). For taproot, conspicuous induction of gene expression was observed for 1-FEH1, 1-FEH2 and VI (**Figure 8**), accompanied by reduced expression of 1-SST and 1-FFT; however, the resulting decline in oligofructans was only modest during 24 h of cold exposure (**Figure 9**).

In a study on chicory hairy roots, it was shown that for their induction 1-FEH1 and 1-FEH2 require different signaling pathways (Kusch et al., 2009), this notion being corroborated by the observation that in a time-course study ABA induction of 1-FEH1 preceded 1-FEH2 induction (Bausewein, 2014; see also **Figure 4**). When comparing the induction kinetics of VI after cold-treatment (**Figure 8**) and ABA-treatment (**Figure 4**), the increased expression is strong but transient in both cases, leading to comparable increases of glucose of young taproots (**Figures 5** and **9**).

## Conclusion

In chicory, optimum growth conditions during the exponential growth phase of taproots and appropriate post-harvest treatment of taproots are key factors for the final goal of high and stable inulin yield (i.e., high degree of polymerization). While the effect of low temperature on inulin degradation at the end of the growing season has been in the focus for many years, the present study has for the first time explored the functional alignment of genes regulating the efficiency of source-to-sink sucrose allocation with those encoding FAZYs in young seedlings, i.e., during the period of exponential taproot growth. The results provide insight into the effects of external cues on fructan profiles (in particular oligofructans), i.e., increase of oligofructans under N-starvation and ABA treatment and decrease of oligofructans under ACC and cold treatments, while gene expression profiles indicate distinct responses for the different treatments affecting the entire plant (leaf blade, petiole and taproot; for a summary of observed gene expression changes see **Supplementary Figures S7**–**S10**). The challenge ahead will be to decipher the coordinate transcriptional control of the relevant gene network. Work on the involved transcription factors (R2R3 MYBs, ERFs) is in progress in the author’s lab.

## Author Contributions

HW: most of the experiments, first draft of the manuscript. AB: method development, ABA treatments. HS: plant cultivation, help with experiments. TS: support of manuscript writing. HZ: support of manuscript writing. KH: conceptional input for experiments. SG: conceptional input for experiments and support for NGS. TR: overall experiment concept, outline of study, finalizing of the manuscript.

## Conflict of Interest Statement

The authors declare that the research was conducted in the absence of any commercial or financial relationships that could be construed as a potential conflict of interest.

## Footnotes

1 http://www.cellnetworks.uni-hd.de/483065/Deep_Sequencing_Core_Facility1

2 http://soap.genomics.org.cn/SOAPdenovo-Trans.html

## Supplementary Material

FIGURE S1 **Relative expression level of reference genes (RPL19 and ACT) in different chicory seedling organs after different treatments (N-starvation, ABA, ACC, cold).** RPL19 expression was normalized for ACT expression in control and N-starved seedlings **(A)**, ABA-treated seedlings **(B)**, ACC-treated seedlings **(C)**, and cold-treated seedlings **(D)**.

FIGURE S2 **Multiple alignment of partial amino acid sequences from three chicory cell wall invertase isoforms (CiCWI1-3) and chicory vacuolar invertase (CiVI).** Conserved β-fructosidase motifs and cysteine-containing catalytic sites are shaded. Red letters indicate the proline residue of the cysteine catalytic site in CWIs, which is substituted by valine in VI. Identical and conserved residues are marked with asterisks and dots/colons, respectively.

FIGURE S3 **Protein sequence alignment (A)** and phylogenetic analysis **(B)** of three chicory sucrose transporter isoforms (CiSUT1-3). The unrooted phylogenetic tree (http://www.phylogeny.fr) displays the relationship between chicory CiSUT1-3 and homologues from *Arabidopsis thaliana*. Accession: AtSUC1 (At1g71880), AtSUC2 (At1g22710), AtSUC3 (At2g02860), AtSUT4 (At1g09960), At SUC5 (At1g71890), AtSUC6 (AT5g43610), AtSUC7 (AT1g66570), AtSUC8 (AT2g14670), AtSUC9 (AT5g06170).

FIGURE S4 **Impact of ABA treatment (72 h) on transcript levels of 1-SST and 1-FFT in taproots. Transcript levels were determined by qPCR and normalized against the expression of RPL19.** Displayed values are means ± SD of three independent experiments. Fold change for ABA treatment was calculated relative to mock samples that were set to 1. Asterisks represent significant differences as determined by Student’s *t*-test (^∗^*P* < 0.05; ^∗∗^*P* < 0.001).

FIGURE S5 **Impact of nitrogen starvation (10 days) on fructan composition in the leaf blade (A)** and the petiole **(B)** of 38-day-old chicory seedlings. Representative sugar profiles of control and N-starved seedlings as obtained by HPAEC-PAD analysis. For abbreviations see **Figure 3**.

FIGURE S6 **Impact of ABA-treatment (24 h) on fructan composition in the petiole of 6-week-old chicory seedlings.** Representative sugar profiles of control and ABA-treated seedlings as obtained by HPAEC-PAD analysis. For abbreviations see **Figure 3**.

FIGURE S7 **Impact of 10-days N-starvation: Cartoon relating observed changes of FAZY transcript levels with transcript levels of CWI and SUT isoforms at whole plant level Upward arrows (red) and downward arrows (blue) indicate significant changes in transcript level as compared to control (^∗^*P* < 0.05; ^∗∗^*P* < 0.001)**.

FIGURE S8 **Impact of 24-h ABA-treatment: Cartoon relating observed changes of FAZY transcript levels with transcript levels of CWI and SUT isoforms at whole plant level.** Upward arrows (red) and downward arrows (blue) indicate significant changes in transcript level as compared to control (^∗^*P* < 0.05; ^∗∗^*P* < 0.001); (a) and (b) refer to up-regulated transcript amounts for 1-SST and 1-FFT, respectively after 72 h of ABA treatment.

FIGURE S9 **Impact of 10-h ACC-treatment (ethylene precursor): Cartoon relating observed changes of FAZY transcript levels with transcript levels of CWI and SUT isoforms at whole plant level.** Upward arrows (red) and downward arrows (blue) indicate significant changes in transcript level as compared to control (^∗^*P* < 0.05; ^∗∗^*P* < 0.001).

FIGURE S10 **Impact of 24-h cold-treatment: Cartoon relating observed changes of FAZY transcript levels with transcript levels of CWI and SUT isoforms at whole plant level.** Upward arrows (red) and downward arrows (blue) indicate significant changes in transcript level as compared to control (^∗^*P* < 0.05; ^∗∗^*P* < 0.001).
