# Supplementary figures and images for: Linking Expression of Fructan Active Enzymes, Cell Wall Invertases and Sucrose Transporters with Fructan Profiles in Growing Taproot of Chicory (Cichorium intybus): Impact of Hormonal and Environmental Cues
Source: Front Plant Sci. 2016 Dec 5;7:1806. doi: 10.3389/fpls.2016.01806 (PMC5136560; doi:10.3389/fpls.2016.01806)

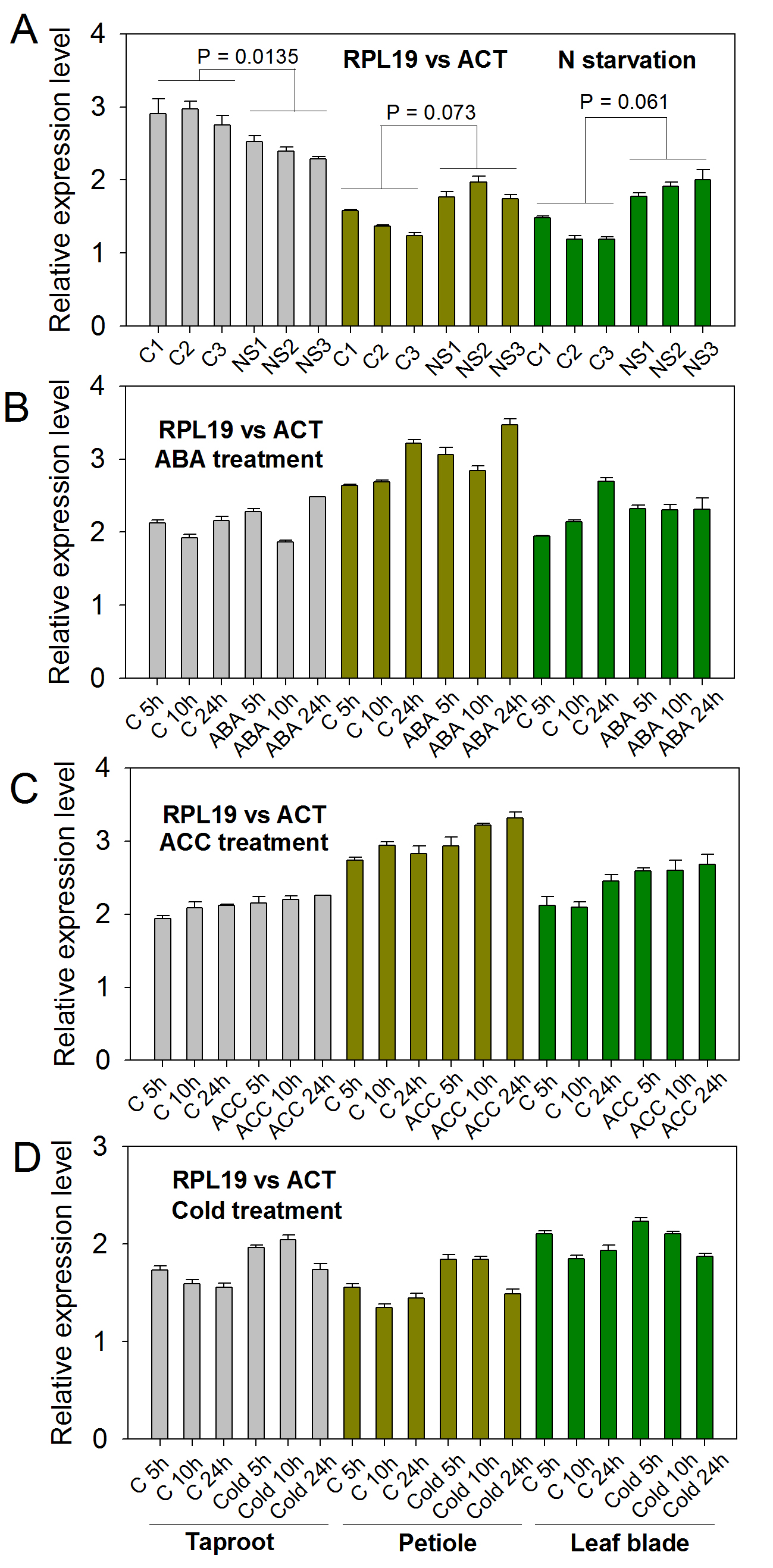

Supplement: FIGURE S1 — Relative expression level of reference genes (RPL19 and ACT) in different chicory seedling organs after different treatments (N-starvation, ABA, ACC, cold). RPL19 expression was normalized for ACT expression in control and N-starved seedlings (A), ABA-treated seedlings (B), ACC-treated seedlings (C), and cold-treated seedlings (D). [file Image_1.JPEG]

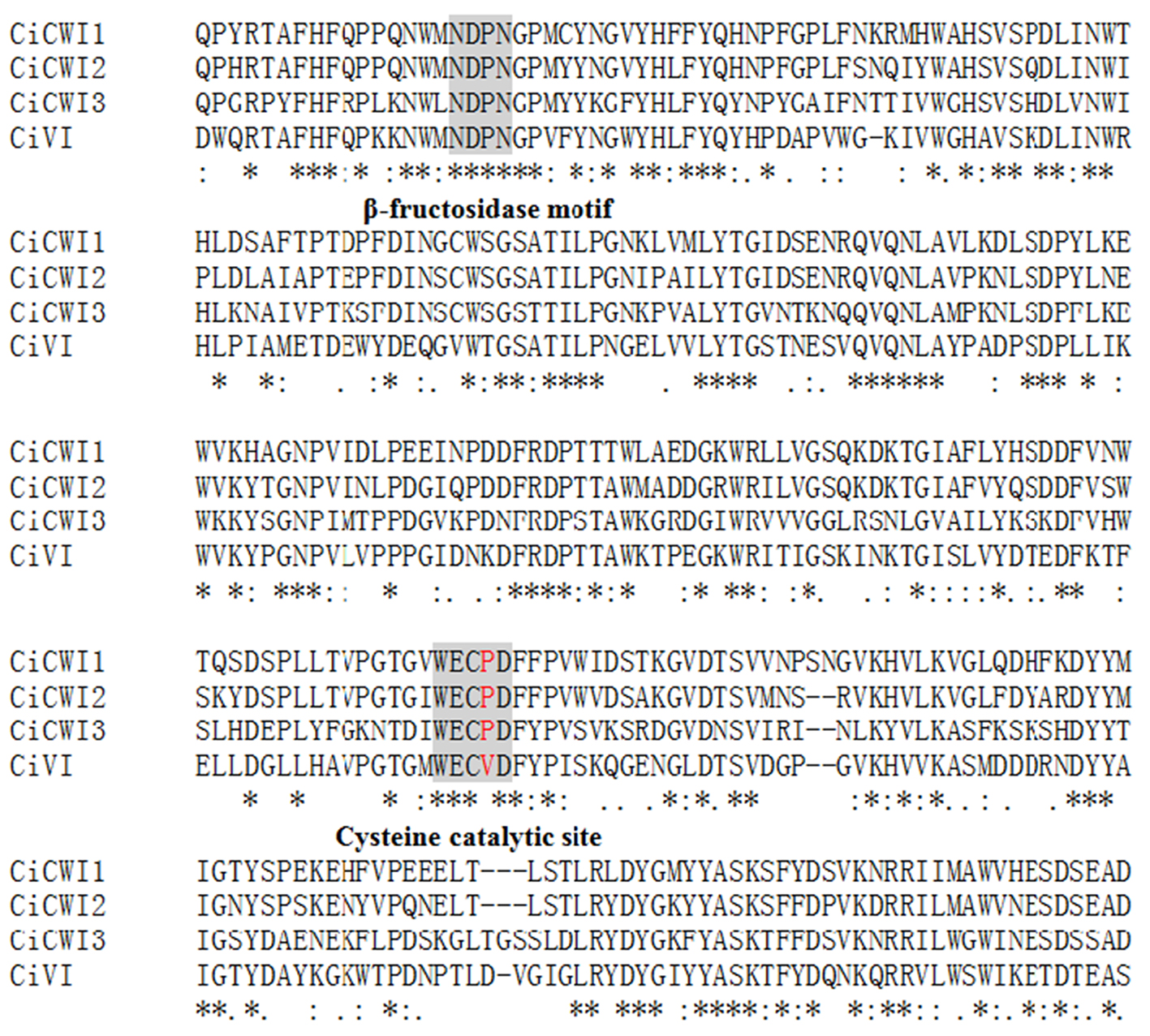

Supplement: FIGURE S2 — Multiple alignment of partial amino acid sequences from three chicory cell wall invertase isoforms (CiCWI1-3) and chicory vacuolar invertase (CiVI). Conserved β-fructosidase motifs and cysteine-containing catalytic sites are shaded. Red letters indicate the proline residue of the cysteine catalytic site in CWIs, which is substituted by valine in VI. Identical and conserved residues are marked with asterisks and dots/colons, respectively. [file Image_2.JPEG]

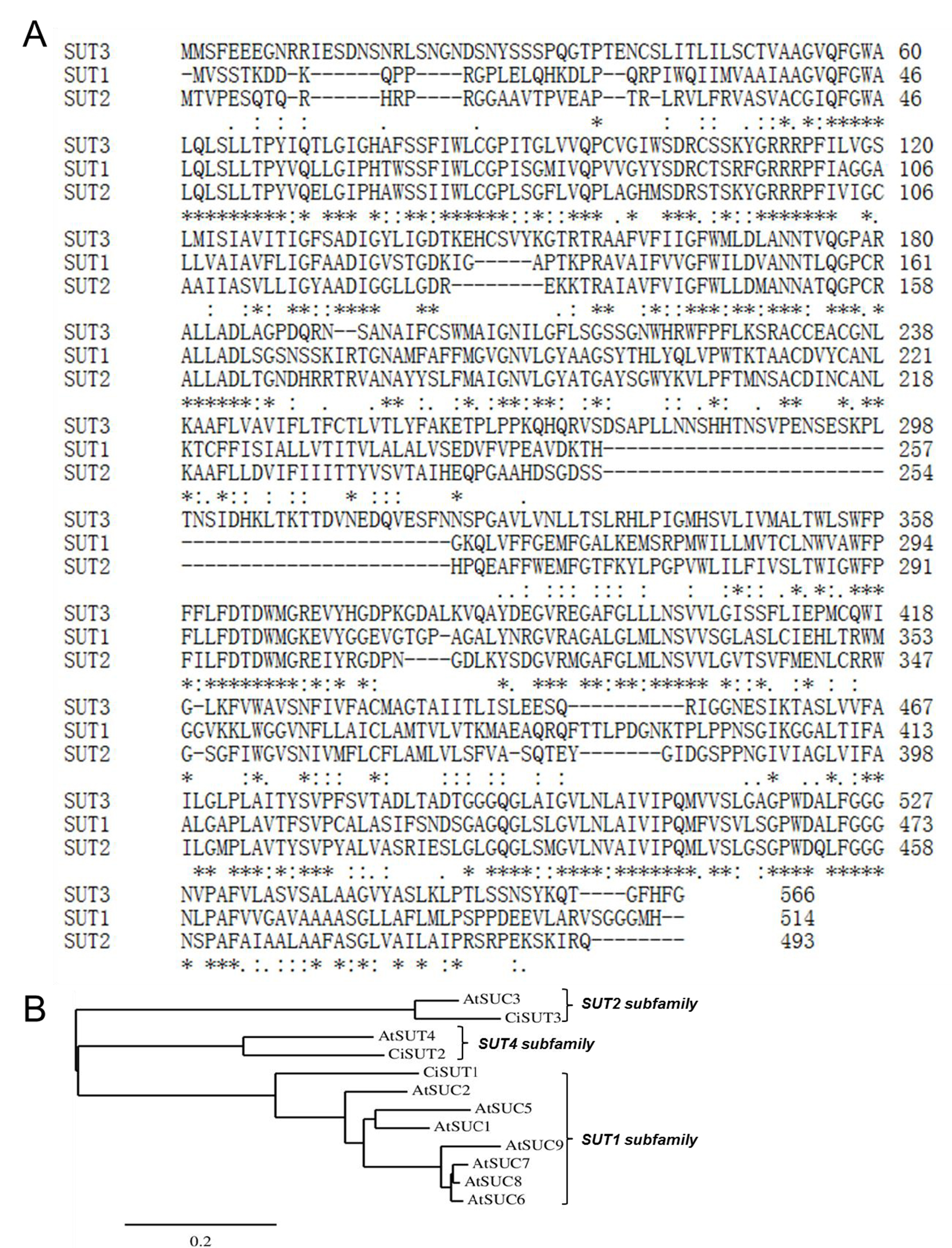

Supplement: FIGURE S3 — Protein sequence alignment (A) and phylogenetic analysis (B) of three chicory sucrose transporter isoforms (CiSUT1-3). The unrooted phylogenetic tree (http://www.phylogeny.fr) displays the relationship between chicory CiSUT1-3 and homologues from Arabidopsis thaliana. Accession: AtSUC1 (At1g71880), AtSUC2 (At1g22710), AtSUC3 (At2g02860), AtSUT4 (At1g09960), At SUC5 (At1g71890), AtSUC6 (AT5g43610), AtSUC7 (AT1g66570), AtSUC8 (AT2g14670), AtSUC9 (AT5g06170). [file Image_3.JPEG]

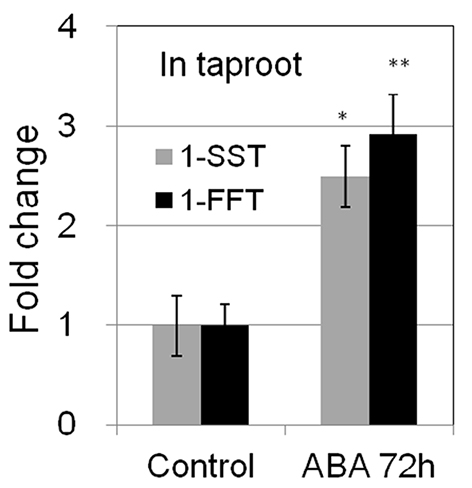

Supplement: FIGURE S4 — Impact of ABA treatment (72 h) on transcript levels of 1-SST and 1-FFT in taproots. Transcript levels were determined by qPCR and normalized against the expression of RPL19. Displayed values are means ± SD of three independent experiments. Fold change for ABA treatment was calculated relative to mock samples that were set to 1. Asterisks represent significant differences as determined by Student’s t-test (∗P < 0.05; ∗∗P < 0.001). [file Image_4.jpg]

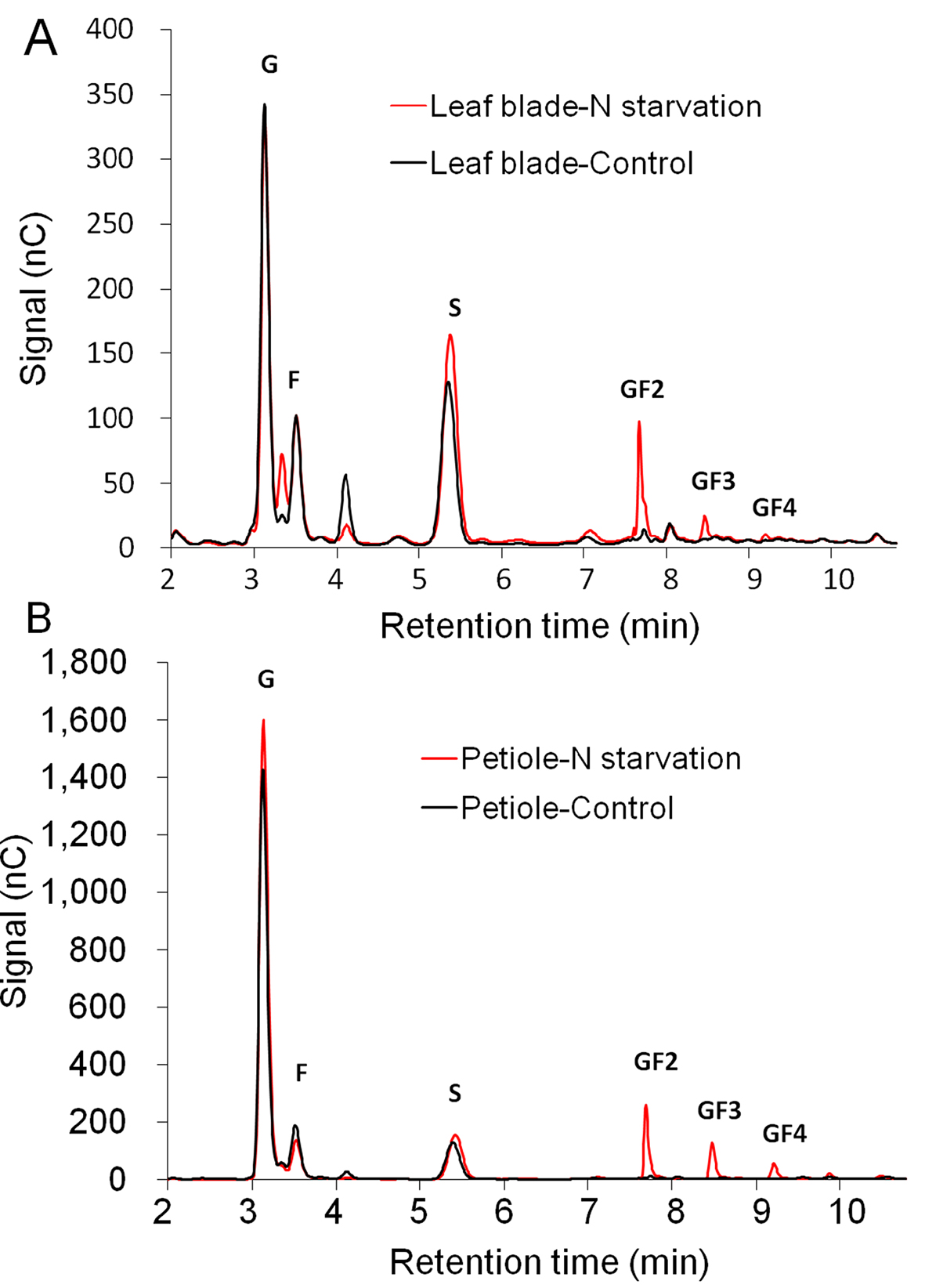

Supplement: FIGURE S5 — Impact of nitrogen starvation (10 days) on fructan composition in the leaf blade (A) and the petiole (B) of 38-day-old chicory seedlings. Representative sugar profiles of control and N-starved seedlings as obtained by HPAEC-PAD analysis. For abbreviations see Figure 3. [file Image_5.jpg]

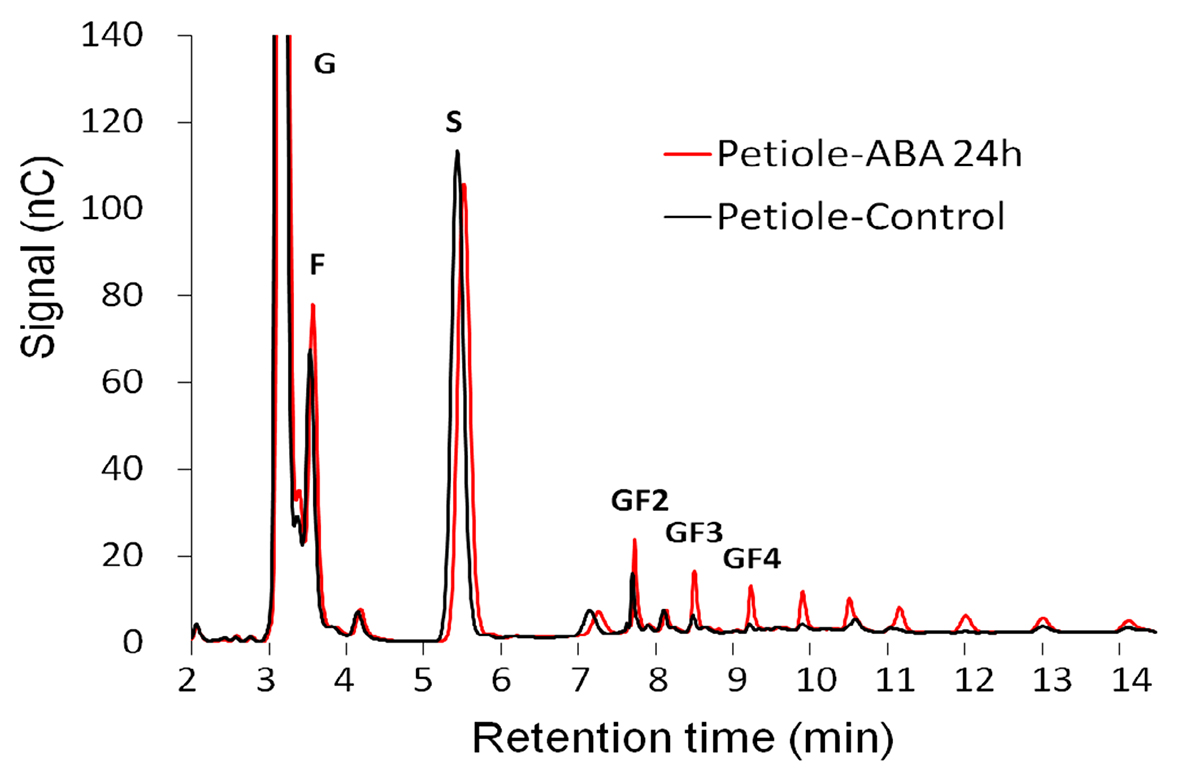

Supplement: FIGURE S6 — Impact of ABA-treatment (24 h) on fructan composition in the petiole of 6-week-old chicory seedlings. Representative sugar profiles of control and ABA-treated seedlings as obtained by HPAEC-PAD analysis. For abbreviations see Figure 3. [file Image_6.jpg]

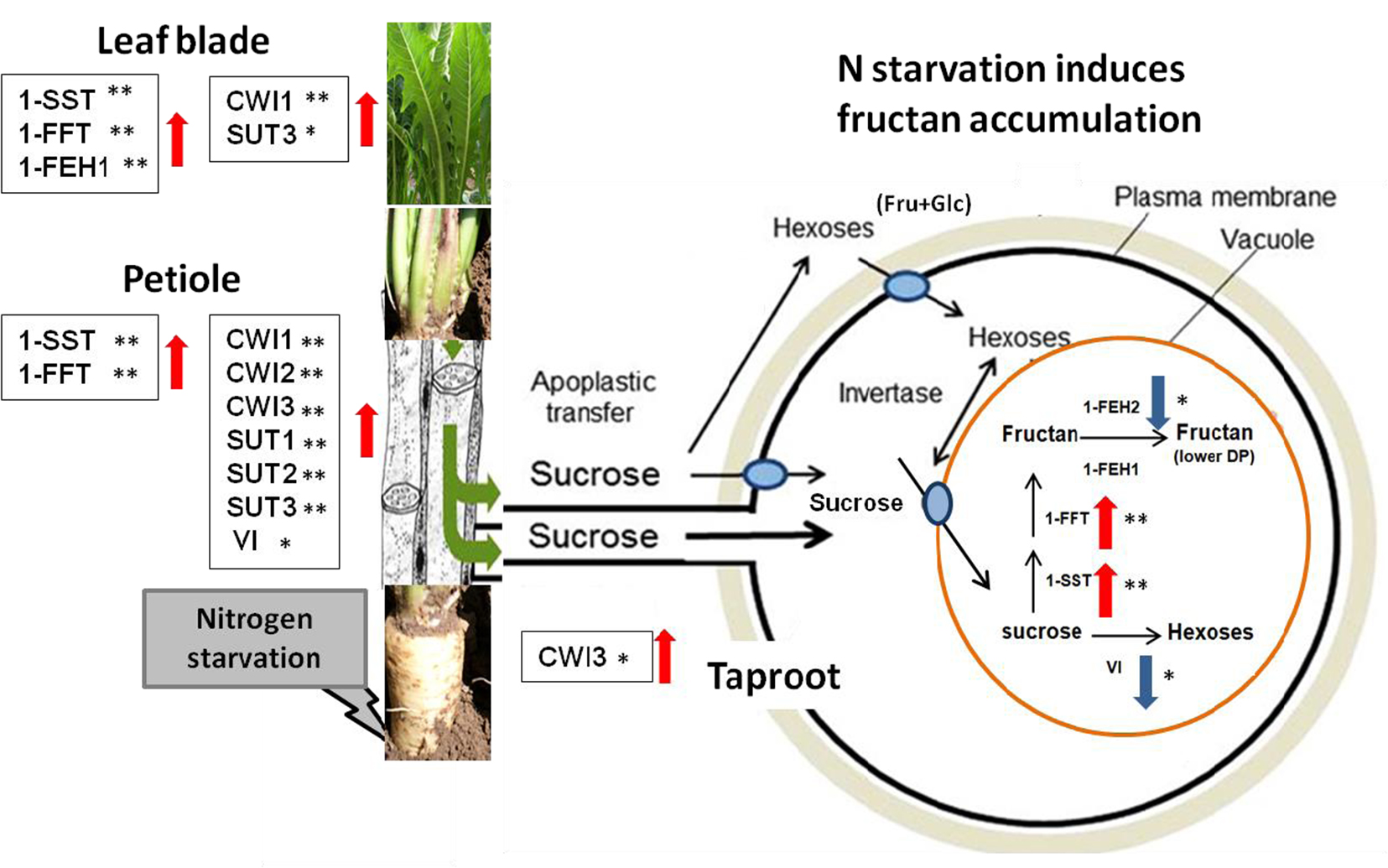

Supplement: FIGURE S7 — Impact of 10-days N-starvation: Cartoon relating observed changes of FAZY transcript levels with transcript levels of CWI and SUT isoforms at whole plant level Upward arrows (red) and downward arrows (blue) indicate significant changes in transcript level as compared to control (∗P < 0.05; ∗∗P < 0.001). [file Image_7.jpg]

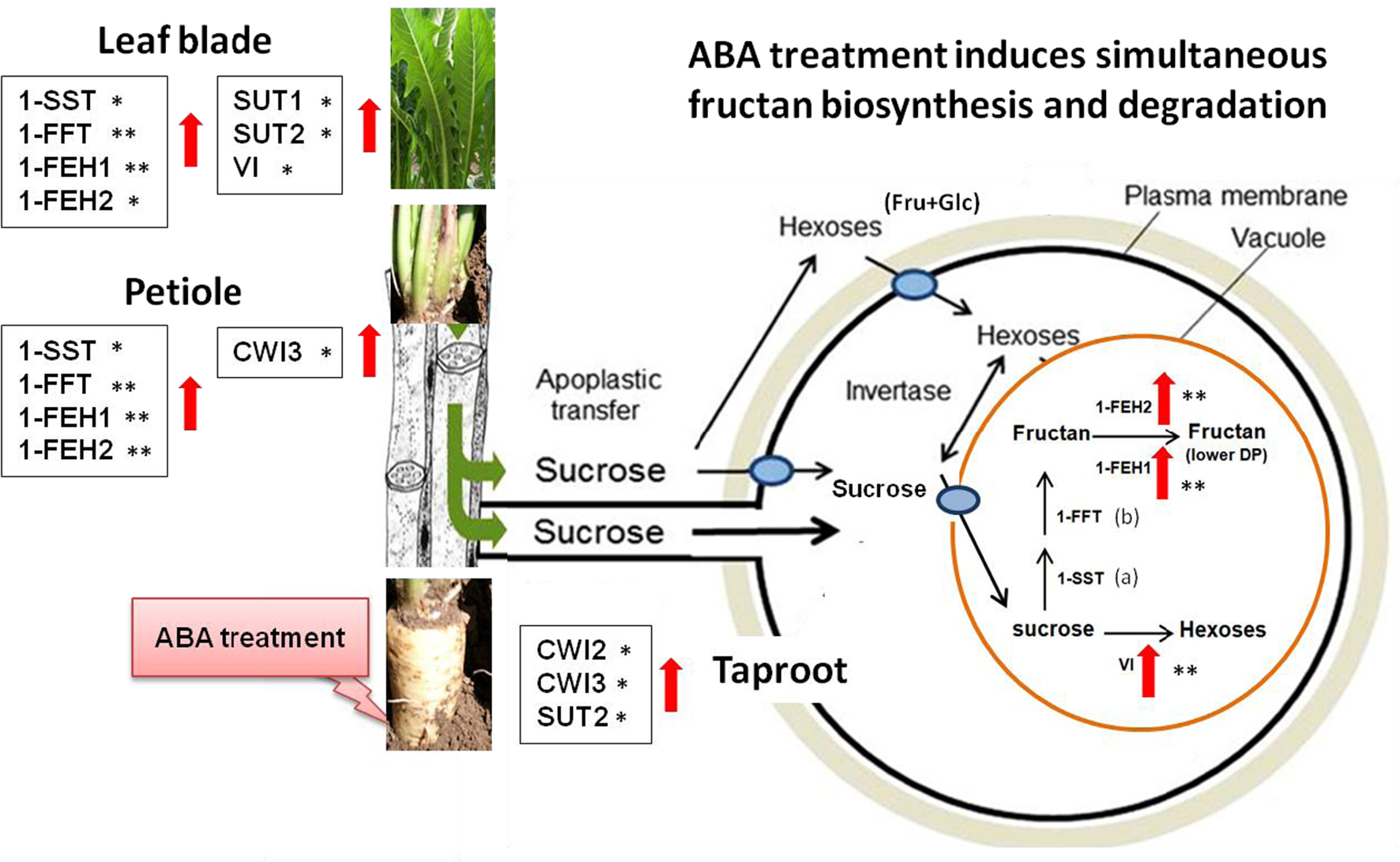

Supplement: FIGURE S8 — Impact of 24-h ABA-treatment: Cartoon relating observed changes of FAZY transcript levels with transcript levels of CWI and SUT isoforms at whole plant level. Upward arrows (red) and downward arrows (blue) indicate significant changes in transcript level as compared to control (∗P < 0.05; ∗∗P < 0.001); (a) and (b) refer to up-regulated transcript amounts for 1-SST and 1-FFT, respectively after 72 h of ABA treatment. [file Image_8.JPEG]

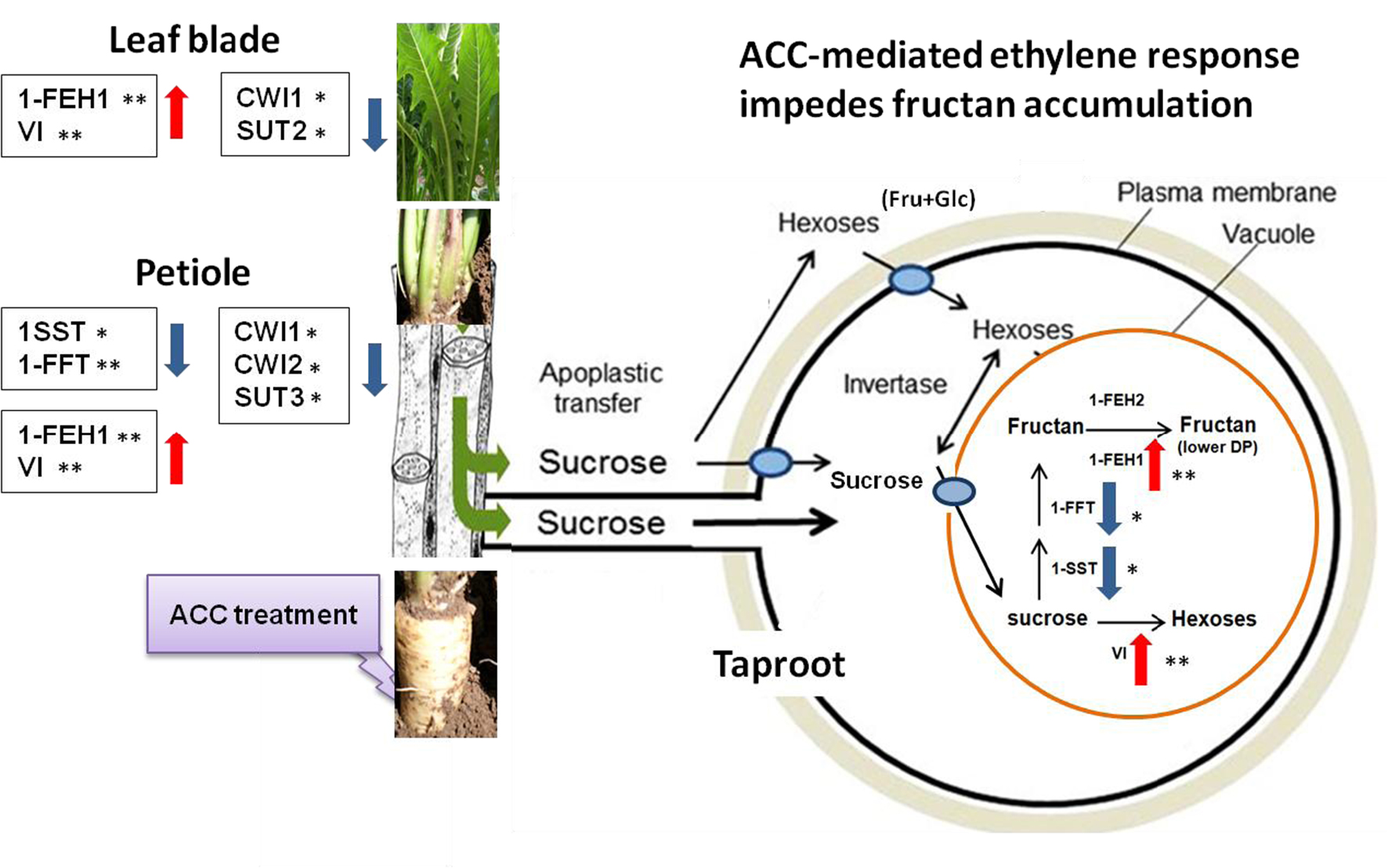

Supplement: FIGURE S9 — Impact of 10-h ACC-treatment (ethylene precursor): Cartoon relating observed changes of FAZY transcript levels with transcript levels of CWI and SUT isoforms at whole plant level. Upward arrows (red) and downward arrows (blue) indicate significant changes in transcript level as compared to control (∗P < 0.05; ∗∗P < 0.001). [file Image_9.JPEG]

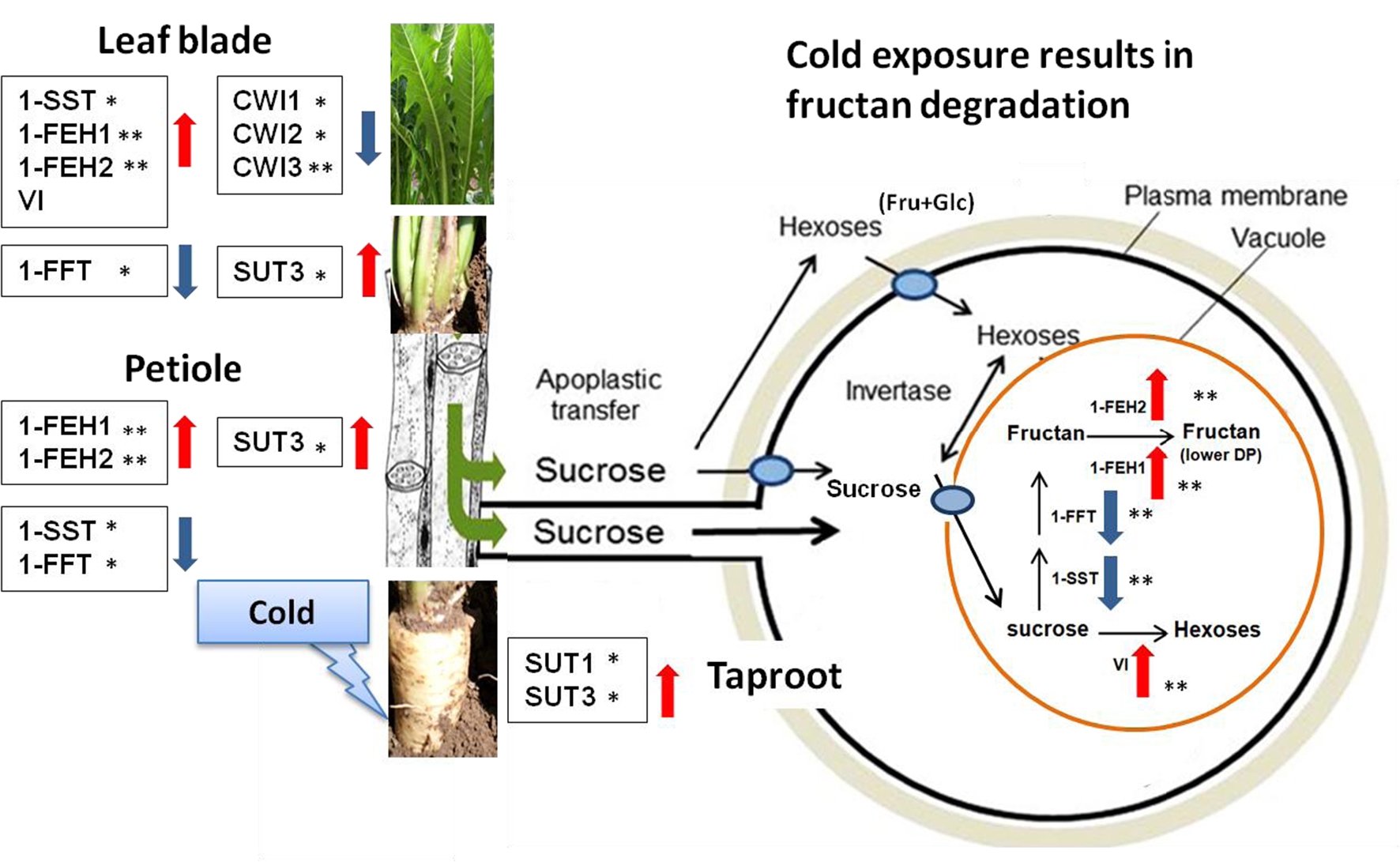

Supplement: FIGURE S10 — Impact of 24-h cold-treatment: Cartoon relating observed changes of FAZY transcript levels with transcript levels of CWI and SUT isoforms at whole plant level. Upward arrows (red) and downward arrows (blue) indicate significant changes in transcript level as compared to control (∗P < 0.05; ∗∗P < 0.001). [file Image_10.JPEG]
