# Supplementary material for: Linking Expression of Fructan Active Enzymes, Cell Wall Invertases and Sucrose Transporters with Fructan Profiles in Growing Taproot of Chicory (Cichorium intybus): Impact of Hormonal and Environmental Cues
Source: Front Plant Sci. 2016 Dec 5;7:1806. doi: 10.3389/fpls.2016.01806 (PMC5136560; doi:10.3389/fpls.2016.01806)
Supplement: Supplementary file 11 [file Table_1.docx]

**Table S1**

The list of primers to amplify the full-length coding sequences (CDS) of genes (CWI2, SUT1&2&3), and the partial coding sequences of CWI1 and CWI3 from a cDNA library prepared from different chicory seedling organs.

| Gene | Description | Primer | Sequences (5‘-3‘) |
| --- | --- | --- | --- |
| CWI1 | Partial CDS 1367 bp | F | atgtctaatcccaacagtacgacgt |
|  |  | R | tgatcactgcacattaccaca |
| CWI2 | Full-length CDS 1701 bp | F | atgaggagaactgccattttct |
|  |  | R | ttatggtgtttggacgttcatt |
| CWI3 | Partial CDS 1042 bp | F | atgaagactattgttgtttgtgtcg |
|  |  | R | cctggattccagcccattctttc |
| SUT1 | Full-length CDS 1545 bp | F | atggtttcctccacgaaaga |
|  |  | R | ctaatgcatccctccacctg |
| SUT2 | Full-length CDS 1482 bp | F | atgacggtgccggaaagt |
|  |  | R | tcattgtcggattttggatttt |
| SUT3 | Full-length CDS 1701 bp | F | atgatgagttttgaggaagaaggt |
|  |  | R | ttatccgaaatgaaaacccg |

**Table S2**

The list of primer pairs for quantitative RT-PCR analysis. Primer efficiency was considered valid when calculated efficiency was between 90% and 110% with 100% as an optimum.

| Gene | Primer | Sequences (5‘-3‘) |
| --- | --- | --- |
| ACT ^(A)^ | F | ccaaatccagctcatcagtcg |
|  | R | tctttcggctccgatggtgat |
| RPL19 ^(B)^ | F | ctgccagcgtcctcaagtg |
|  | R | cattgggatcaagccaaacct |
| 1-SST | F | ccaacaaccatcagggaggag |
|  | R | agcaacggagctgtgaacgt |
| 1-FFT | F | cggctacgcagttggacatag |
|  | R | ctcgtggtgcaaccgtattca |
| 1-FEH1 | F | gataaacgatcctaacggac |
|  | R | gtagaacagatggtaaactcca |
| 1-FEH2 | F | taaagacttgaaagaacaaagtg |
|  | R | tgcaccataacttgtcgtgtcg |
| VI | F | gctcactcgttccactcaacttg |
|  | R | ggctccgttcaatcgttccac |
| CWI1 | F | caaagaatgggtcaagcacgctg |
|  | R | caagccaggtggtggtcgga |
| CWI2 | F | gaccctgtgaaagaccgaagaatc |
|  | R | tcccgaccatcctttagcgatat |
| CWI3 | F | atgccattgttccaaccaagtcgt |
|  | R | tgactccagtgtacaacgcgact |
| SUT1 | F | tcttacactcacctctaccaactc |
|  | R | atcctccgacaccaacgcta |
| SUT2 | F | caactcgctgggaaattgttatcc |
|  | R | acggtgcctttgtgtttgactt |
| SUT3 | F | acattggtgacactctactttgc |
|  | R | tgtggtgagaattgttaagaagcg |
| Susy | F | gttcacaatggcgagactggac |
|  | R | attccttccttctgtcaccacct |

Note: (A) and (B) are valid chicory reference genes from (Maroufi et al., 2010) and (van Arkel et al., 2012) respectively.
